# The Ethic of Access: An AIDS Activist Won Public Access to Experimental Therapies, and This Must Now Extend to Psychedelics for Mental Illness

**DOI:** 10.3389/fpsyt.2021.680626

**Published:** 2021-07-05

**Authors:** Morgan Campbell, Monnica T. Williams

**Affiliations:** ^1^Delaware Division of Substance Abuse and Mental Health, Department of Psychiatry, New Castle, DE, United States; ^2^School of Psychology, University of Ottawa, Ottawa, ON, Canada; ^3^Department of Cellular and Molecular Medicine, University of Ottawa, Ottawa, ON, Canada

**Keywords:** access, advocacy, empowerment, psychedelics, discrimination, stigma, ethics

## Abstract

If patients with mental illnesses are to be treated fairly in comparison with other categories of patients, they must be given access to promising experimental therapies, including psychedelics. The right of early access to promising therapies was advanced as an ethical principle by activist Larry Kramer during the AIDS pandemic, and has now largely been adopted by the medical establishment. Patients are regularly granted access to experimental drugs for many illness categories, such as cancer and infectious diseases. The need for expanded access is especially relevant during evolving crises like the AIDS and the coronavirus pandemics. In contrast to non-psychiatric branches of medicine, psychiatry has failed to expedite access to promising drugs in the face of public health emergencies, psychological crises, the wishes of many patients, and the needs of the community. Psychiatry must catch up to the rest of medicine and allow the preferences of patients for access to guide policy and law regarding unapproved medications like psychedelics.

## Advances in Medical Ethics

There are many beneficiaries of medical research, although ultimately research is done in the name of patients, for their benefit, and at their risk. Therefore, end-users of therapies should have the authority to empower doctors and scientists, not the other way around. Within psychiatry, there has been a failure to grant access to experimental drugs in the face of dire need and the wishes of many patients and their loved ones. Lessons from AIDS Coalition to Unleash Power (ACT UP) should embarrass us into action.

### Context

In the past, the greatest threat to patients was that doctors would set aside human rights and dignity in the name of aggressive advances in science. Nazi scientists in World War Two and American doctors in the Tuskegee Syphilis Study made the problem clear to the whole world. The scientific community largely addressed these concerns by codifying principles for ethical research on human subjects in the Nuremberg Code (1947), the Declaration of Helsinki (1964), and the Belmont Report (1978) (1).

As a result of these codes and established norms, we now do a better job of protecting patients from being exploited, but hidden costs of an ethics built on restraining science emerge when sick multitudes race against time for life-saving cures. Consider playwright-turned patients' rights activist Larry Kramer and ACT UP. In 1988, at the height of the AIDS pandemic, gay men in Kramer's community were dying of AIDS without chances to try cutting-edge treatments. Kramer saw that clinical trial protocols were slow-walking the introduction of new therapies. He denounced Dr. Anthony Fauci—then as now, the director of the National Institute of Allergy and Infectious Diseases —in the *San Francisco Examiner*: “There are more AIDS victims dead because you didn't test drugs on them than because you did” (2). Kramer used offensive terms to shock and embarrass a complacent medical establishment into acknowledging that bigotry was delaying access: “Why? Because this disease is happening to faggots, n^***^ers, spics, junkies, and hookers” (3). ACT UP mobilized protests and established an underground market for experimental drugs. Consequently, the medical establishment buckled. Patients were included in FDA advisory panels, and access was expanded for experimental drugs. This applied not only to therapies for AIDS, but also for cancer, infectious diseases, and other non-psychiatric illnesses. Dr. Fauci summarized the impact in Kramer's obituary: “He totally transformed the relationship between activism and the scientific, regulatory, and government community” (4). Kramer and ACT UP created a path for patients to transition from passive consumers of therapies to active agents in medical history.

This path endures. During the coronavirus pandemic, widespread public oversight of drug development facilitated public buy-in, which was crucial for bold innovation. Stephen M. Hahn, Commissioner of Food and Drugs for the Food and Drug Administration wrote, “No time in recent memory has shown as bright a light on the work of the FDA review staff as the COVID-19 pandemic…. Transparency regarding the FDA's thinking about scientific data needed to support safe and effective vaccines will help build public confidence in the FDA's evaluation process” (5). With the public engaged, the FDA was able to implement the Coronavirus Treatment Acceleration Program, which “uses every available method to move new treatments to patients as quickly as possible, while at the same time finding out whether they are helpful or harmful” (6). The public gained early access to therapies such as convalescent plasma and direct-acting antivirals through Mayo Clinic-led expanded access protocols. Emergency use authorization for the first vaccine was granted by the FDA on December 11, 2020 (7).

## People with Mental Illnesses are Left Out

In the present mental health crisis, unlike the AIDS and coronavirus pandemics, drug developers are failing to serve stigmatized populations in a time of great need.

The world is experiencing a dire mental health emergency. The global burden of mental health and substance use disorders increased 37.6% between 1990 and 2010. Mental health and substance use disorders were the top contributor of years lived with disease worldwide in 2010 (8). Along with suffering and despair inherent in PTSD, treatment-resistant depression and psychological trauma, these conditions increase risk for chronic physical health problems and suicide. Suicides have increased by 35% in the past two decades. For girls and women, the increase is 50% (9, 10). Suicide rates have also increased among people of color, with a stunning 139% increase among Native American women (11). The coronavirus has accelerated trends of escalating mood disorders. During the pandemic, the proportion of US adults who reported symptoms of anxiety and depression rose from one in 10 (January to June, 2019) to four in 10 during the pandemic (April, 2020 to March, 2021) (12). There was a 21% increase in selective serotonin reputable inhibitor (SSRI) prescriptions in the USA from February 16, 2020 to March 15, 2020 (13). A cross-sectional study of nearly 190 million emergency department (ED) visits found increases in ED visits for all mental health conditions and suicide attempts. Opioid overdoses were 29% higher in 2020 than before the pandemic (14). Eighteen to 24 year-olds are most heavily impacted, with one quarter of this population reporting that they have seriously considered suicide during the pandemic (15). These burdens are even greater for people who are part of racialized communities. People of color with mental illness face a double stigma, and COVID-19 has disproportionately burdened people of color (16, 17). The increased rates of illness and death have especially impacted people of color, and subsequently led to more distress stemming from grief, loneliness, frustration, and economic hardship.

Of the few standard therapies that exist for addiction, depression, and PTSD, none are adequate to address the mental health crisis. For example, the two medications approved to treat PTSD by the FDA, paroxetine hydrochloride and sertraline hydrochloride, have only small to moderate effect sizes compared to placebo (18). Selective serotonin reuptake inhibitors (SSRIs), the mainstay treatments for major depression, fail to help the majority of depressed patients achieve remission (19, 20). In fact, SSRIs may work no better than placebo for mild depression (21). Antipsychotics do not help with schizophrenia's negative symptoms like anhedonia and isolation, which are the symptoms that matter most to patients (22). Antidepressants and antipsychotics did not arise from an aggressive search for molecular targets. Drugs with action against depression, mania, and psychosis were discovered by chance in the course of testing drugs for tuberculosis (drugs targeting the serotonin system), anesthesia (dopamine agonist antipsychotics), and epilepsy (valproic acid). Since the advent of selective serotonin reuptake inhibitors in 1988, few psychiatric medications with novel action have been brought to patients. Notable exceptions include orexin antagonists for insomnia. Recent therapeutic breakthroughs for cancer, COPD, and coronary artery disease vastly exceed advances in psychiatry (23–25).

Pharmaceutical investment in psychiatric therapies continues to remain low compared to the burden of diseases, with 3.1 dollars invested per 1,000 in disease burden for schizophrenia, 1.8 for major depression, and 0.4 for bipolar disorder, compared to 9.4 for chronic obstructive pulmonary disease (COPD), 7.6 for diabetes, and 75.5 for cancer (26).

Like AIDS sufferers, mental health patients face stigma and discrimination, which are barriers in mobilizing drug development and expediting access. Access to new therapies is also lacking because symptoms of mental illnesses are not always outwardly apparent, and symptoms such as lack of motivation and disorganization can prevent patients from effectively advocating for themselves.

## Hope and Possibilities

For mental health, the ancient medications we now call psychedelics have been used for generations by indigenous people in therapeutic contexts. The Mazatec people of Mexico gave us psilocybin, adherents of the Bwiti religion in Gabon discovered ibogaine, and the Shipibo people of the Amazon basin developed ayahuasca (27). Clinical trials at Imperial College London and Johns Hopkins suggest that psilocybin is more effective in remediating depression than any currently approved therapies (28, 29), and also suggest psilocybin's impressive action against addiction, obsessive compulsive disorder, along with other mental health conditions (30). Sixty eight perecntage of patients with PTSD who received therapy with 3,4-methylenedioxymethamphetamine (MDMA) in a recent phase 2 trial no longer met criteria for PTSD 1 year after their treatment (28). Open-label clinical studies and surveys demonstrate that suicide and suicide rates are greatly reduced by psychedelic medications (31–33).

In the early 1950s, the psychedelic compound LSD had already begun to show promise for remediating various mental illnesses in clinical settings. Despite this, research into psychedelics was halted in the latter half of the twentieth century in response to their recreational use by club-goers and their prominent role in the left-wing counterculture of the Vietnam era. The therapeutic molecules were stigmatized along with the population of patients who stood to benefit. This may explain why it has taken so long to provide mentally ill patients with access, even in comparison to AIDS patients who also contend against stigma. Even now that many legal restrictions have been lifted and research has been allowed, there are significant barriers to this research, including lack of institutional support and difficulty in obtaining funding. Of note, funding for psychedelic research has come almost entirely from private sources (34, 35).

The Multidisciplinary Association for Psychedelic Studies (MAPS) has been at the forefront of advancing the research on therapies for PTSD using MDMA. MDMA is an entactogen phenethylamine that bears similarities to classical psychedelics like LSD and psilocybin in its subjective effects, mechanism of action, and stigmatized status (28, 36). While their multisite Phase 3 trials were underway, MAPS began an ambitious training program for therapists in anticipation of the vast demand for MDMA treatment through the FDA Expanded Access Program. Expanded Access, also known as “compassionate use,” is a process that makes promising experimental drugs available to patients who have not responded to conventional treatments. MAPS submitted their application to the FDA in January of 2019, enrolled over 700 therapists in their clinician training program, and registered over 120 clinics to be a part of the expanded access roll-out. MAPS initially anticipated that 250 to 1,000 people would enroll in the Expanded Access Program throughout the US. The psychedelic research community was stunned when the FDA approved the application but limited the number of patients who could participate to a mere 50, forcing MAPS to scale back its plans and leaving hundreds of desperate patients to languish on waiting lists (37, 38). Through a similar mechanism, MAPS has applied to Health Canada for 20 patients to be treated with MDMA at a Vancouver clinic (39). In another swipe at accessibility, the FDA erected highly restrictive conditions on Expanded Access therapists. In addition to requiring two therapists to be present for the full duration of all psychedelic sessions, the FDA is now requiring that the lead facilitator be an MD or PhD, and that there be doctor on site the whole time rather than just on call, which is making the already high cost of treatment so high that it would be inaccessible to any but the wealthiest. MAPS is objecting to the enrollment limits and the other restrictions placed by the FDA and has engaged lawyers to help resolve the dispute.

Demand for psychedelic medications from people seeking help for their mental illnesses has led to underground use, in a way that parallels how patients took risks to obtain unapproved medications during the AIDS pandemic (40, 41). This underground use has been most perilous for people of color, who face greater stigma and legal risks due to the War on Drugs (42). Nonetheless, some are even using psychedelics to self-treat traumatization due to racism (43).

Despite the wishes of many patients, the Johns Hopkins team argue that risk mitigation should be paramount for psychedelics. They write, “More randomized clinical trials are necessary to replicate and extend the promising findings of open-label trials, and to better understand contraindications and therapeutic risks.” According to them, “We owe it to the next generation of researchers and clinicians and to millions of patients who may benefit from these treatments that no exceptions be made to standards of research” (35). These researchers' caution is understandable in light the history of psychedelic research being shut down in the past, the difficulties in restarting this research, and the overly optimistic notions among some that these medications may act as cure-alls.

Nevertheless, the AIDS and coronavirus pandemics expose a need to update standards of research in psychiatry. Along with the hard-won principle that doctors must, “first do no harm,” we must honor the public's need to access new therapies that are developed not only for the most visible crises, but also for the mental health crisis, where intense suffering can be hidden from public view.

Open questions include how to amplify the voices of patients regarding experimental therapies like psychedelics, how to implement early access, how to educate the public about this option once it exists, and how to ensure equitable access for multiple marginalized groups. A model of political engagement like ACT UP may not work for patients whose symptoms include lack of motivation and will, and who are at risk for re-traumatization. The authors are exploring an entirely patient-led counterpart to traditional academic peer review, which allows diverse patient communities to provide meaningful input into therapies that result from trials.

As we consider solutions, Larry Kramer's plea for faster access to AIDS medications echoes to remind us that for many patients, time is limited: “We are in the middle of a f^**^king plague!” (44).

## Data Availability Statement

The original contributions presented in the study are included in the article/supplementary material, further inquiries can be directed to the corresponding author/s.

## Author Contributions

MC developed the concept of the article and discussed it with MW who added ideas. MC wrote the initial draft. MW edited and approved the final version for submission. All authors contributed to the article and approved the submitted version.

## Conflict of Interest

The authors declare that the research was conducted in the absence of any commercial or financial relationships that could be construed as a potential conflict of interest.
